# The importance of placental lipid metabolism across gestation in obese and non-obese pregnancies

**DOI:** 10.1042/CS20220657

**Published:** 2023-01-03

**Authors:** Kyle M. Siemers, Michelle L. Baack

**Affiliations:** 1 University of South Dakota, Sanford School of Medicine, Sioux Falls, SD, U.S.A.; 2 Sanford Research, Environmental Influences on Health and Disease Group, Sioux Falls, SD, U.S.A.

**Keywords:** fatty acid binding proteins, fatty acid oxidation, lipid metabolism, obesity, Placenta, pregnancy

## Abstract

In this commentary, we highlight a new study by Bidne and colleagues that identifies changes in placental lipids and lipid metabolic enzymes that happen not only in the context of parental obesity but also from as early as 4 weeks of gestation. Their assessment of lipid and enzyme content demonstrates a feasible approach to untangling the complexities of metabolic pathologies that impact the lifelong health of both parent and child.

The importance of studying placental lipid metabolism cannot be overemphasized because it not only influences pregnancy outcomes but also fetal growth, development, and life-long health [1]. A growing body of evidence demonstrates that the assumption of free diffusion of maternal fatty acids to the developing fetus is no longer accepted. The placenta converts circulating maternal lipids to free fatty acids (FFAs) for uptake and processing by trophoblast cells, which use them to meet their own metabolic demands, to produce hormones for pregnancy maintenance, and to transfer them to the developing fetus [2,3]. Robust lipid uptake and metabolism early in gestation is vital to meeting the high energetic demands needed to simultaneously grow the placenta and develop embryonic organ systems. Late in gestation the human fetus requires more lipids for neurodevelopment and growth than any other land mammal, so as pregnancy progresses, metabolic adaptations in the mother and placenta uniquely support increasing lipid transport and biomagnification of essential long chain polyunsaturated fatty acids (LCPUFA) in the last trimester [2]. These particular fatty acids are important components of phospholipid membranes where they serve as local mediators of metabolism, inflammation, immune function, platelet aggregation, signal transduction, neurotransmission, and neurogenesis that is vital for the developing fetal brain and retina [4]. Because LCPUFA cannot be synthesized *de novo*, the fetus relies on increasing placental transport, especially during the last trimester when peak *in utero* accretion can surpass maternal intake to support rapid fetal brain growth [4]. For these reasons, placental lipid uptake and metabolism is a critical, highly-regulated, and surprisingly dynamic process across gestation.

Beyond the need to understand placental lipid transport and metabolism in uncomplicated pregnancies, there is an even more urgent need to identify maladaptations that occur in common conditions such as obesity as well as to understand the downstream consequences for the parent–placenta–fetal triad. The prevalence of obesity (body mass index ≥ 30 kg/m^2^) is increasing worldwide to impact approximately 40% of women of reproductive age in the United States [5]. Obesity is associated with poor pregnancy outcomes [6] and serious lifelong health risks not only for the parent [7], but also for the child. Maternal obesity increases the risk for the child being born large for gestational age (LGA) [8–10], with birth defects [11], relative insulin resistance [12], and increased adiposity [10,13], which translates to a higher lifetime risk for developing Type 2 diabetes, obesity, cardiometabolic disease, cognitive impairment, and mood disorders [10,14]. Increasing evidence demonstrates that disturbances in placental lipid metabolism play an important role in the pathogenesis of these adverse outcomes, specifically via oxidative stress and variable fatty acid transport to the fetus [15–18].

As the fields of reproductive health and developmental origins of health and disease look to the placenta for answers regarding the role of fuels in driving pregnancy and fetal outcomes [1], it will be essential to overcome gaps in knowledge about adaptations in placental lipid metabolism across critical windows of gestational risk. The use of animal models has been fundamental to understanding this but translating findings to humans is challenging. This is primarily because of difficulty obtaining samples at various stages of pregnancy. Indeed, most studies focus on the term, postpartum placenta which reflects only the endpoint and fails to identify critical windows for intervention or compounded exposures across fetal development. Birth defects or malformations, common morbidities associated with obese pregnancy, develop very early in the first trimester and are reportedly mediated by abnormal lipid metabolism and excess reactive oxygen species production [19]. Studying second trimester placentae is key to understanding the role of lipid metabolism in placental maladaptation and the high rate of stillbirths found in obese pregnancies [20–22]. Yet, accessing first and second trimester placentas for research is very challenging and fraught with confounding.

In this issue of *Clinical Science*, Bidne et al. have been able to surmount this challenge to examine human placentas and cord blood ranging from 4 to 40 weeks gestation [23]. Their study builds upon previous work detailing placental nutrient transporters across gestation [24] by quantifying lipid fractions, transporters, and metabolic pathways in first, second, and third trimester placenta, then comparing these levels between obese and non-obese pregnant women. Admittedly, the study has limitations including unreported differences in total lipid content, individual placental cell populations, and understandable limitations in details about whether placentas collected at 4–24 weeks gestation were complicated by fetal anomalies or would have gone on to cause gestational diabetes or preeclampsia. Regardless, the work provides valuable insight on obesity-associated differences in fatty acid transport at multiple stages of gestation. Specifically, the authors identify the abundance of various phospholipid, lysophospholipid, and triacylglycerol species, in addition to levels of proteins important for lipid uptake, metabolism and synthesis, particularly those involved in β-oxidation, intracellular trafficking, storage, and the Kennedy and Lands’ pathways as illustrated in Figure 1. Although the authors conclude that there are only limited obesity-related differences, detailing the changes in placental lipid content in the first and second trimester is a necessary step needed to understand how obesity during these early critical windows can cause compounding consequences in placental and fetal development as well as metabolism to affect pregnancy outcomes.

**Figure 1:**
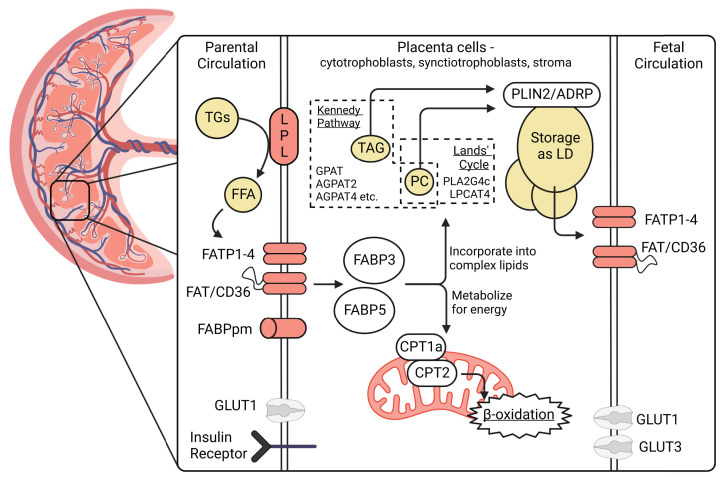
Model of fatty acid transport for energy and complex lipid production in placental tissue Circulating triglycerides (TGs) in the parental circulation are metabolized into FFAs by lipases like lipoprotein lipase (LPL) at the placenta. The FFAs are able to be transported into placental cells by a variety of fatty acid transport proteins (FATP), fatty acid translocase (FAT/CD36), and plasma membrane-bound fatty acid binding proteins (FABPpm), alongside other crucial fuels like glucose in response to physiologic alterations in insulin and plasma lipids in pregnancy. Intracellular fatty acid binding proteins (FABP3 and 5) shuttle fatty acids toward incorporation into phospholipids or toward energy production through β-oxidation in the mitochondria (facilitated by the carnitine shuttle system's CPT1a and CPT2). Triacylglycerols (TAG) and well as phospholipids like phosphatidylcholine (PC) are synthesized within the placenta for their storage as lipid droplets, facilitated by PLIN2/adipophilin (ADRP), and their mobilization to be transported into fetal circulation. Acyl-transferase enzymes like glycerol-3-phosphate acyltransferase (GPAT) and 1-acylglycerol-3-phosphate-O-acyltransferases (AGPAT2 and AGPAT4) in the Kennedy Pathway and phospholipase A2 (PLA2G4c) and lysophosphatidylcholine acyltransferase (LPCAT4) in the Lands’ cycle remodel the fatty acid species being transported to the fetus. These elements of lipid metabolism in the placenta can be studied over gestation as well as between control and obese pregnancies to determine the mechanisms driving this important element of fuel transport, as demonstrated by Bidne et al. [23] in this issue *of Clinical Science*. Created with BioRender.com.

One could feasibly use Bidne et al.’s approach to gather a breadth of knowledge about how other disease states, toxins, or environmental exposures could disrupt the parent-placenta-fetal triad to contribute to other reported adverse pregnancy or programming consequences. It would also be possible to use their methods to compare placenta supporting males compared with those supporting females to understand sex-specific differences in fetal growth and programming. Answering fundamental questions about the role of lipid metabolism in the parent-placenta-fetal triad across gestation will certainly set a foundation needed to identify mechanisms and critical windows for intervention that impact numerous pregnancies and children worldwide.

## Data Availability

Data sharing is not applicable to the paper.

## Abbreviations

FFA: free fatty acids
LCPUFA: long chain polyunsaturated fatty acids
LGA: large for gestational age
LPL: lipoprotein lipase
PC: phosphatidylcholine
TAG: triacylglycerol
TG: triglyceride
